# Extended high-frequency audiometry in older adults: hearing thresholds

**DOI:** 10.1007/s00405-026-10098-y

**Published:** 2026-03-20

**Authors:** Nikol Gottfriedová, Michaela Škerková, Eva Mrázková, Ľubica Argalášová, Jana Jurkovičová, Kateřina Němček, Hana Tomášková

**Affiliations:** 1 Department of Epidemiology and Public Health, Faculty of Medicine, University of Ostrava, Syllabova 19, Ostrava, Czech Republic; 2 Department of Occupational Safety and Processes, Faculty of Safety Engineering, VŠB Technical University Ostrava, Ostrava, Czech Republic; 3 Center for Hearing and Balance Disorders, Ostrava, Czech Republic; 4 Department of Otorhinolaryngology and Head and Neck Surgery, Havířov Hospital and Clinic, Havířov, Czech Republic; 5 Institute of Hygiene, Faculty of Medicine, Comenius University in Bratislava, Bratislava, Slovakia; 6 Department of Physiology and Pathophysiology, Faculty of Medicine, University of Ostrava, Ostrava, Czech Republic; 7 Institute of Public Health in Ostrava, Ostrava, Czech Republic

**Keywords:** Extended high-frequency audiometry, Pure-tone audiometry, Hearing thresholds, Elderly

## Abstract

**Purpose:**

This research aimed to determine hearing thresholds in healthy older adults (≥ 60 years of age) using extended high-frequency audiometry (EHFA).

**Methods:**

The study included 334 individuals aged ≥ 60 years with no history of hearing problems before the age of 60 and no occupational noise exposure. Inclusion criteria further required normal findings on otoscopy and tympanometry, as well as an audiometric response at at least three extended high frequencies (EHFs) in at least one ear. Participants were categorized by sex and age (60–69 years; 70–79 years; 80 + years). All underwent conventional pure-tone audiometry with a frequency range of 0.125–8 kHz and EHFA with a range of 9–14 kHz. The results were measured in dB HL and evaluated separately for men and women as well as for each age category. Statistical testing was performed at the 5% significance level.

**Results:**

Significant differences between older and younger age groups were observed for both men and women. Hearing thresholds of women were higher in some low frequencies (0.125–0.5 kHz), whereas in men, greater hearing loss was observed at frequencies from 3 kHz onwards. The audiometric response exceeded 93% of tested ears in both sexes and across all age categories up to 11.25 kHz; at 12.5 kHz and 14 kHz, the response declined.

**Conclusion:**

With increasing age and higher frequency, the hearing thresholds also increased and the audiometric response declined. The results contribute to a better understanding of extended high-frequency hearing thresholds in older adults.

## Introduction

Hearing loss and deafness constitute a global problem. World Health Organisation (WHO) reported that approx. 430 million individuals worldwide are affected by disabling hearing loss requiring rehabilitation [1] and this number is expected to grow to 700 million by 2050 [1]. Approximately 30% of elderly individuals ≥ 60 years of age are estimated to be affected [2]. Age-related hearing loss (ARHL) and noise-induced hearing loss (NIHL) are the most common types of hearing impairment. ARHL typically begins to clinically manifest at the age of approx. 60 years and can lead to issues with spoken language comprehension and, in effect, social isolation of affected individuals [3]. Hearing loss is also considered a risk factor for dementia development [3, 4]. The negative consequences of hearing loss are, however, largely preventable by early diagnosis, which highlights the importance of its early detection.

Extended high-frequency audiometry (EHFA) is a potentially highly suitable and sensitive method of hearing loss detection [5], providing an examination of hearing thresholds at frequencies exceeding the standard range of conventional audiometry, i.e., > 8 kHz. Human ears can capture frequencies of up to 20 kHz, with the damage to the hearing thresholds being observable at the highest frequencies first [5]. The EN ISO 7029:2017 standard defines median hearing threshold values at audiometric frequencies of 0.125–8 kHz; at higher frequencies of 9–12.5 kHz, the values may be considered indicative. No thresholds are given for frequencies > 12.5 kHz [6]. In the oldest age categories, the expected high-frequency median hearing thresholds are also subject to uncertainty due to the relative lack of source data [6].

This research aimed to determine hearing thresholds in adults ≥ 60 years of age unexposed to occupational noise at frequencies of 9–14 kHz and, thus, to help improve knowledge of EHF hearing thresholds for older adults.

## Materials and methods

The data were collected over a two-year period at a cooperating ear, nose, and throat (ENT) clinic. Participants aged 60–91 years, living in the same industrial region, were selected from the facility’s database (*n* = 334; 113 men; 221 women). Inclusion criteria were (i) age ≥ 60 years; (ii) no personal history of hearing impairment before 60 years of age; (iii) no history of risk occupational noise exposure (A-weighted equivalent sound pressure level L_Aeq,8 h_ ≥ 80 dB); (iv) normal findings on otoscopy and tympanometry; (v) audiometric response at at least three EHFs in at least one ear, and (vi) cognitive ability to provide personal medical history and to undergo the audiometry examination. Subsequently, individuals with presbycusis degree classified as disabling hearing loss according to the WHO’s Grades of hearing impairment [7] were excluded, as well as individuals with an asymmetrical hearing loss with a difference between the left and right ear of > 15 dB. The presence of slight hearing impairment (i.e., pure tone average (PTA) at 500, 1000, 2000, 4000 Hz in the better ear ≤ 40 dB) was not disqualifying from participation [7].

### Data collection and processing

Qualified medical staff of the ENT clinic conducted a multi-step examination. Medical history was obtained through a structured interview. First, personal history was taken with an emphasis on subjective hearing assessment, history of hearing problems, middle ear inflammation, and other past and present diseases. This was followed by employment history, and family history with a particular focus on the occurrence of hearing loss in the family.

After that, the participants underwent tympanometry (Madsen Zodiac Diagnostic, Type 1096 device), conventional pure-tone audiometry (CA; 0.125–8 kHz) and extended high-frequency audiometry (EHFA; 9–14 kHz). Hearing was examined in both the right and left ears of all participants (i.e., 668 ears). The examination took place in a soundproof audiometric booth, using the Madsen Astera 2 audiometer with a dedicated Sennheiser HDA300 headset. All instruments were calibrated before the measurements. The measurements were performed according to EN ISO 8253-1:2010 ‘Acoustics – Audiometric test methods’ [8] (last revised and validated in 2021), and to EN ISO 266:1997 ‘Acoustics – Preferred frequencies’ [9] (last revised and validated in 2023). Unless the participant reported a clear difference in hearing between the ears, measurement started with the right ear; where differences were reported, the ear with better hearing was measured first. The sound intensity was increased in 5 dB steps until the participant reported hearing the sound. Then, the intensity was stepwise reduced until the participant ceased to signal hearing the tone. The hearing threshold was determined as the level at which hearing was repeatedly confirmed by the participant. The results for the right and left ears were recorded in audiograms with decibel hearing level (dB HL).

### Statistical processing

The participants were classified into three categories according to their age: (i) 60–69; (ii) 70–79; (iii) 80 + years. The median values in dB HL at all mentioned frequencies were calculated for each age category in both men and women. Measurements from both ears were used for determining medians, i.e., the number of analyzed ears was double that of the number of participants. Statistical significance analyses for the evaluation of hearing thresholds were performed using the Mann-Whitney non-parametric test, while chi-square or Fisher’s exact test were used for the analysis of audiometric response as appropriate. All tests were performed at a 5% significance level. The Stata software (version 13) was employed for all analyses.

## Results

### Study population

In all, 334 adults (668 ears) from an industrial region were included in the study. Of that, 113 were men (33.8%) with a mean age of 68.5 years (SD = 7.6; min = 60; max = 91) and 221 were women (66.2%), mean age of 69.7 years (SD = 7.1; min = 60; max = 87). No statistical difference between age in men and women was detected (*p* = 0.063). Of the three age categories (60–69; 70–79; 80 + years), the 60–69 years category was the most represented (178 participants), while the 80 + category (43 participants) was the least abundant.

### Audiometric response

At CA frequencies (0.125–8 kHz), all participants (and all ears) responded to all frequencies regardless of sex and age group. At EHFs (9–11.25 kHz), both men and women showed more than 90% audiometric response in all age groups (Table 1). Response decreased with increasing age and with increasing frequency (Table 1).

Compared with younger women, those aged 70–79 years showed a significantly lower proportion of ears with an audiometric response from 10 kHz onward. The same was observed in women aged 80 + years compared with those aged 60–69 years at frequencies of 12.5 and 14 kHz. In men aged 70–79 years, the proportion of ears with an audiometric response was significantly lower than in younger men at 12.5 kHz. This pattern was also evident in men aged 80 + years compared with those aged 60–69 years at 12.5 and 14 kHz.

**Table 1:** Percentage of ears with audiometric response in men and women in individual age groups

			Men			Women		
	Age group (years)		60–69	70–79	80+	60–69	70–79	80+
	Number of ears		132	64	30	224	162	56
Frequency [Hz]		6,000	100%	100%	100%	99.6%	100%	100%
		8,000	100%	100%	100%	99.6%	100%	100%
		9,000	97.7%	96.9%	96.7%	99.6%	98.1%	98.2%
		10,000	97.7%	93.8%	96.7%	100%	96.9%▲	98.2%
		11,250	95.5%*♂*	93.8%	96.7%	99.6%*♂*	95.1%▲	96.4%
		12,500	86.4%*♂*	75.0%▲	63.3%*♂*▲	93.8%*♂*	78.4%▲	83.9%*♂*▲
		14,000	47.0%*♂*	35.9%	20.0%▲	71.0%*♂*	43.8%▲	21.4%▲■

▲= statistically significantly different from the 60–69 age group of the same sex (*p* < 0.05); ■= statistically significantly different from the 70–79 age group of the same sex (*p* < 0.05); ♂= statistically significantly poorer audiometric response in men compared to women of the same age group (*p* < 0.05). Fisher exact test or chi-square test.

Table 1 shows the percentage of ears with an audiometric response at individual frequencies. Statistically significant differences are indicated in the table using the following symbols: (i) the triangle symbol denotes a significantly lower proportion of ears with an audiometric response compared with the 60–69 age group; (ii) the square symbol denotes a lower proportion of responsive ears compared with the 70–79 age category; (iii) the male symbol (♂) indicates a statistically significantly lower proportion of ears with an audiometric response in men compared with women within the same age category.

**Fig. 1:**
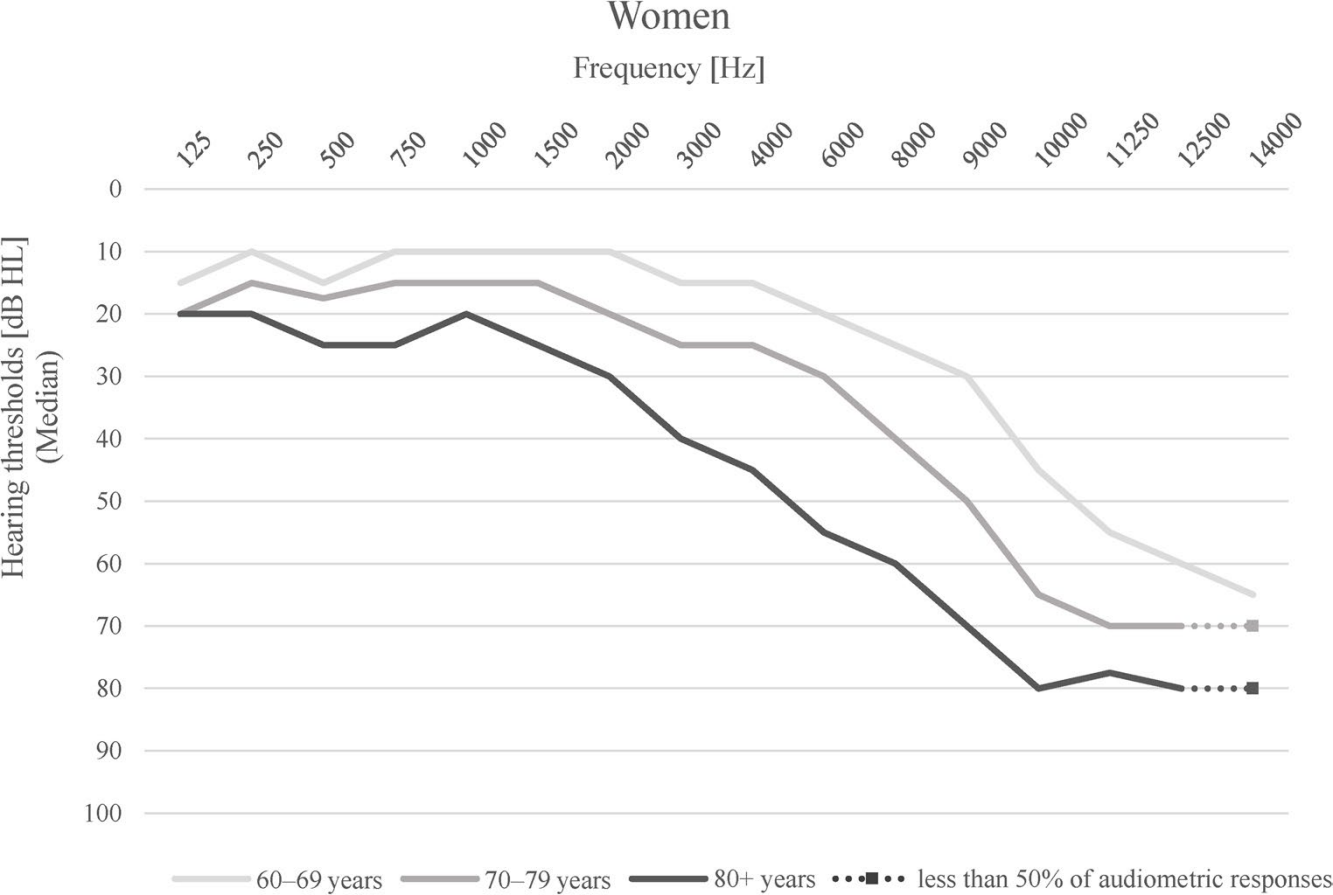
Median hearing thresholds for individual age groups and frequencies in older women

**Fig. 2:**
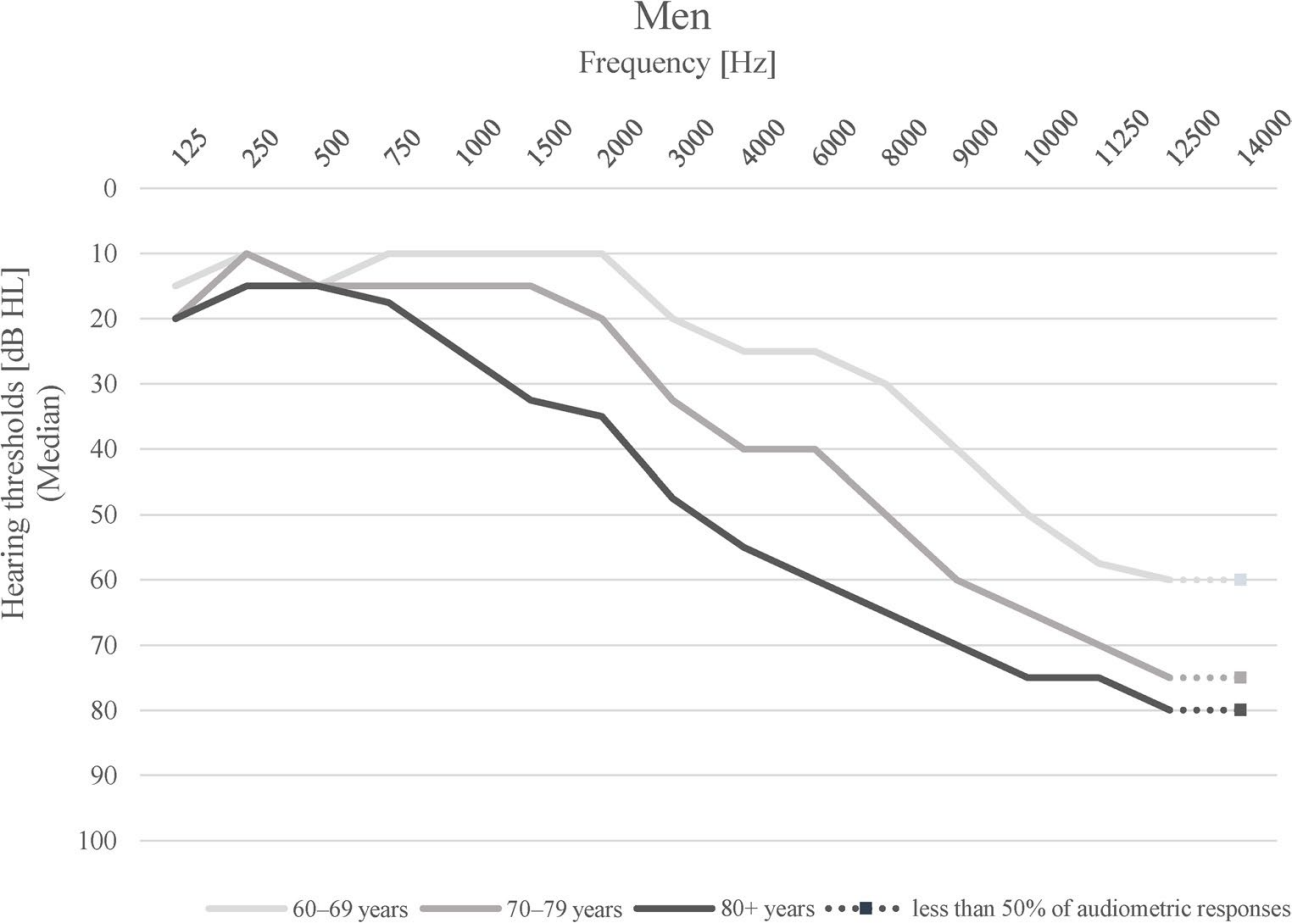
Median hearing thresholds for individual age groups and frequencies in older men

At the highest frequency of 14 kHz, with the exception of the youngest female group (60–69 years), the audiometric response was observed in less than half of examined ears in both sexes. Figures 1 and 2 show the audiograms, with this frequency being depicted using a dotted line.

### Comparison of hearing thresholds in individual age groups

General trends in hearing thresholds of both sexes were similar; increasing age and frequency were associated with increasing (i.e., worsening) hearing thresholds (Figs. 1 and 2).

Hearing thresholds (dB HL) were statistically significantly higher in older age groups compared to younger ones at nearly all frequencies (*p* < 0.001) regardless of sex (Table 2). Hearing thresholds significantly differed between sexes at some low (≤ 0.5 kHz) as well as higher (≥ 3 kHz) frequencies. Significantly greater hearing loss was observed in women than in men at some low frequencies (0.125 kHz and 0.5 kHz). On the other hand, men had significantly worse hearing thresholds than women at frequencies of 3–9 kHz (Table 2).

**Table 2:** Hearing thresholds and significant differences between age groups and sexes

	Hearing thresholds [dB]; median values (min; max)							
		Men (226 ears)				Women (452 ears)		
Age group			60–69 years	70–79 years	80 + years	60–69 years	70–79 years	80 + years
Number of ears			132 ears	64 ears	30 ears	224 ears	162 ears	56 ears
Conventional audiometry (Frequencies – Hz)	125		15 (5; 25)♀	20 (5; 45)▲	20 (10; 45)▲	15 (0; 30)♀	20 (5; 35)▲	20 (10; 45)▲■
	250		10 (0; 30)	10 (0; 25)▲	15 (5; 35)▲■	10 (0; 25)	15 (0; 30)▲	20 (10; 45)▲■
	500		15 (0; 30)	15 (5; 35)▲	15 (5; 40)♀▲	15 (0; 25)	17.5 (0; 35)▲	25 (10; 55)♀▲■
	750		10 (0; 30)	15 (5; 30)▲	17.5 (5; 45)▲	10 (0; 30)	15 (0; 40)▲	25 (10; 50)▲■
	1,000		10 (0; 30)	15 (0; 40)▲	25 (5; 45)▲■	10 (0; 30)	15 (5; 35)▲	20 (5; 45)▲■
	1,500		10 (0; 35)	15 (0; 35)▲	32.5 (10; 55)▲■	10 (0; 30)	15 (0; 40)▲	25 (10; 55)▲■
	2,000		10 (0; 30)	20 (0; 45)▲	35 (5; 65)▲■	10 (0; 45)	20 (0; 55)▲	30 (10; 50)▲■
	3,000		20 (0; 50)♂	32.5 (15; 50)♂▲	47.5 (25; 65)♂▲■	15 (0; 40)♂	25 (0; 65)♂▲	40 (10; 65)♂▲■
	4,000		25 (0; 55)♂	40 (15; 60)♂▲	55 (30; 75)♂▲■	15 (0; 55)♂	25 (0; 65)♂▲	45 (10; 65)♂▲■
	6,000		25 (0; 65)♂	40 (0; 70)♂▲	60 (30; 75)♂▲■	20 (0; 85)♂	30 (0; 65)♂▲	55 (5; 85)♂▲■
	8,000		30 (0; 75)♂	50 (10; 85)▲	65 (50; 95)▲■	25 (0; 90)♂	40 (10; 85)▲	60 (15; 90)▲■
Extended high-frequency audiometry (Frequencies – Hz)	9,000		40 (0; 80)♂	60 (10; 95)♂▲	70 (55; 85)▲■	30 (0; 80)♂	50 (0; 90)♂▲	70 (30; 100)▲■
	10,000		50 (5; 100)	65 (20; 95)▲	75 (55; 95)▲■	45 (0; 90)	65 (5; 90)▲	80 (30; 100)▲■
	11,250		57.5 (10; 90)	70 (25; 100)▲	75 (60; 100)▲■	55 (0; 90)	70 (5; 100)▲	77.5 (35; 100)▲■
	12,500		60 (5; 95)	75 (45; 90)▲	80 (70; 90)▲	60 (5; 95)	70 (0; 95)▲	80 (40; 100)▲■
	14,000		60 (0; 85)	75 (45; 85)▲	80 (75; 85)▲ ■	65 (0; 85)	70 (10; 85)▲	80 (40; 85)▲■

▲= statistically significantly different from the 60–69 age group of the same sex (*p* < 0.05); ■= statistically significantly different from the 70–79 age group of the same sex (*p* < 0.05); ♂= statistically significantly worse hearing thresholds in men compared to women of the same age group (*p* < 0.05); ♀= statistically significantly worse hearing thresholds in women compared to men of the same age group (*p* < 0.05). Non-parametric Mann-Whitney test

Table 2 presents an overview of median hearing thresholds (dB HL), together with the minimum and maximum observed values (min; max). Statistically significant differences between groups are indicated by the following symbols: (i) the triangle symbol indicates significantly higher hearing thresholds within the same sex compared with the 60–69 age group; (ii) the square symbol indicates the same comparison with the 70–79 age group; (iii) the male or female symbol (♂; ♀) indicates significantly higher hearing thresholds compared with the opposite sex within the same age category. Statistical comparisons of hearing thresholds were performed using the nonparametric Mann–Whitney test.

## Discussion

This study aimed to investigate hearing thresholds in individuals aged ≥ 60 years classified by sex and age (60–69; 70–79; 80 + years). The present results contribute to the current knowledge on extended high frequencies, as the EN ISO 7029:2017 [6] standard provides only informative values for this frequency range, with an upper limit of 12.5 kHz. As the standard continues to develop, the data presented in this study may contribute to the establishment of normative hearing threshold values in the extended high-frequency range (i.e., frequencies above 8 kHz).

As expected, the trends observed in the present study were similar in men and women, with hearing thresholds increasing with age and frequency (Figs. 1 and 2). This pattern is consistent with trends reported in otologically healthy individuals by Stenklev and Laukli [10]. They recorded low audiometric response at a frequency of 12.5 kHz in both men and women older than 80 years of age. In our study, the outcome in this category at the same frequency was better – over 63% in men and 83% in women. Low response rates at the frequency of 14 kHz, on the other hand, were very consistent in our study and that by Stenklev & Laukli [10] as well as that by Lee et al. [11]. Present study revealed a significant difference in the number of ears with audiometric response between men and women in the 60–69 age group, with women showing significantly higher responsiveness at some high frequencies (≥ 11.25 kHz; Table 1).

In terms of maximum measured values, audiometric responses in several patients in our cohort were recorded only at the highest (i.e., 100 dB HL) used intensity level (Table 2). Stenklev & Laukli reported median hearing thresholds as poor as 110 dB in participants aged 60 years and older [10]. Rodríguez Valiente et al. used noise levels of up to 120 dB in their study (and used the same value for statistical purposes also in nonresponsive ears) [12]. A similar method of statistical evaluation was employed by Lee et al., who used a value of 115 dB for nonresponsive ears [11]. In a 2024 study, Luengrungrus et al. measured hearing thresholds of individuals ≤ 70 years of age, classified into 10-years intervals [13]. In the > 60–70 years age group, their hearing thresholds at frequencies of 9 kHz and 10 kHz were similar to ours, while at higher frequencies of 11.2–14 kHz, hearing thresholds determined in their study were higher than in ours [13]. These differences can possibly be attributed to sociocultural differences between study populations.

One of the inclusion criteria for the present study was sufficient cognitive ability to undergo pure-tone audiometric testing. Individuals with dementia have a reduced ability to successfully complete pure-tone audiometry [14]; Bott et al. reported that only 56–59% of adults with dementia were able to complete pure-tone audiometric testing successfully [14]. In addition, greater hearing loss has been observed in patients with dementia [15–18], and similar findings have also been reported for extended high frequencies (EHFs). Waechter et al. found an association between hearing thresholds at EHFs (10–16 kHz) and everyday cognitive performance, with worse EHF thresholds being associated with poorer performance [19]. However, no association was observed between the presence of tinnitus and cognitive performance [19]. Helfer et al. examined associations between EHF hearing thresholds (9–12 kHz) and cognitive abilities in a cohort of middle-aged and older adults and reported that higher (worse) hearing thresholds at EHFs were associated with poorer cognitive performance in older adults [20].

Participants with a history of occupational noise exposure were not included in the study. Age-related hearing loss can be first detected at the highest frequencies [5, 21, 22] and occupational hearing loss resulting from exposure to high levels of noise at the workplace also typically manifests initially at high frequencies [23–25]. A meta-analysis [26] showed a significant association between occupational noise exposure and hearing thresholds, with medium effect sizes at 9 and 11.2 kHz and large effect sizes at 10, 12, 14, and 16 kHz [26]. A significant association was also observed for recreational noise exposure, with effects at 12, 12.5, and 16 kHz [26]. In the case of individuals exposed to occupational noise, EHFA was shown to be a useful tool for the early detection of noise-induced hearing loss [24, 25], even when hearing thresholds at frequencies ≤ 8 kHz remain within the normal range [26]. In the case of recreational noise, the findings are not yet consistent. Some studies have reported an association between hearing thresholds at EHFs and long-term use of personal listening devices [27], whereas others have found no association between recreational noise exposure and EHF hearing thresholds [28].

Although other methods for the detection of hearing loss in older adults have been successfully used before, such as evoked otoacoustic emissions [29], smartphone-based tests [30], or standardized questionnaires (e.g. HHI-S) [31], pure-tone audiometry remains the gold standard for hearing loss examination across age categories. The results of our study can contribute to the current knowledge about EHFA in older adults and improve the understanding of EHF hearing thresholds in this population.

## Limitation of the study

The lower number of male participants is a limitation of our study. One of the factors that have contributed to this difference is a higher frequency of occupational noise exposure among men compared to women in the industrial region where our study took place. As such noise exposure was one of the exclusion criteria, this fact led to the rejection of many potential male participants. In this context, the fact that all respondents were from the industrial region burdened also with traffic noise can be considered as another limitation of the study. Personal medication history was retrieved by questioning the patients, which can also be considered a limitation of this study as we cannot fully exclude the use of ototoxic drugs. However, no participant explicitly reported the use of medications with documented ototoxicity during the medical history at the time of the examination. Another limitation that should be considered is that participants were not randomly selected but were recruited from the database of a single facility, and data collection was not conducted across multiple ENT clinics. However, a key advantage of conducting all examinations at a single ENT clinic is that they were performed by the same qualified medical staff, using the same equipment, and under identical conditions for all participants. A general limitation in older adults is their typically poorer hearing at EHFs due to age-related hearing loss. This limitation is also evident in our study, in which median hearing threshold values at the highest frequency (14 kHz) were determined in fewer than 50% of the examined ears across most age categories, with the exception of the youngest female group, in which an audiometric response at 14 kHz was detected in 71% of ears.

## Conclusion

This study aimed to determine hearing thresholds on conventional frequencies and extended high frequencies (EHFs) in men and women aged ≥ 60 years, thus, to help improve knowledge of EHFA in this population. Increasing age and frequency were associated with increasing (i.e., worsening) hearing thresholds and declining audiometric response rates. Importantly, we found that despite slight age-related hearing loss, which is a common symptom of the physiological aging of the auditory system, adults ≥ 60 years of age showed a > 93% audiometric response up to a frequency of 11.25 kHz. Further research on extended high-frequency hearing thresholds in these age groups is needed to establish normative values.

## Data Availability

The study materials and the details of all analyses are available from the corresponding authors upon reasonable request.
